# MicroRNA-638 inhibits cell growth and tubule formation by suppressing VEGFA expression in human Ewing sarcoma cells

**DOI:** 10.1042/BSR20171017

**Published:** 2018-01-19

**Authors:** Xin Zhou, Jiajun Chen, Qianren Xiao, Tengyu Wang, Yu Yu, Bo Li, Gaohai Shao, Yunyun Li, Zhongzu Zhang

**Affiliations:** 1 Department of Orthopedics, The Yongchuan Hospital of Chongqing Medical University, Chongqing 402160, P.R. China; 2 Department of Pain Management, the Affiliated Hospital of Jinggangshan University, Ji’an, Jiangxi 343000, P.R. China; 3 Department of Gynecology and Obstetrics, The Yongchuan Hospital of Chongqing Medical University, Chongqing 402160, P.R. China

**Keywords:** Angiogenesis, Ewing Sarcoma, miR-638, VEGFA

## Abstract

Ewing sarcoma (EWS) is a kind of aggressive tumor of bone and soft tissues, which most occurring in children and adolescents. MicroRNAs (miRNAs) perform essential function in the progression and development of EWS, while the putative role of miR-638 in EWS remains uncertain. Accordingly, we detected the expression of miR-638 and explored its putative biological effects on the malignant phenotype of EWS cells. As expected, miR-638 was significantly down-regulated in EWS cells. Moreover, overexpression of miR-638 suppressed cell growth, induced cell apoptosis, and inhibited tubule formation of EWS cells *in vitro*. Among the putative target genes of miR-638 predicted by the miRNA target prediction tools, vascular endothelial cell growth factor A (VEGFA) attracted out attention most. The luciferase reporter assays reaffirmed that VEGFA was a targeted gene of miR-638 in EWS cells. Furthermore, miR-638 suppressed the mRNA and protein level of VEGFA, and restored the expression of VEGFA reversed the suppressed effects of miR-638 in EWS cells. Taken together, the results suggested that miR-638 might perform tumor suppressive effects in EWS, which might be mediated, at least partially, through suppressing the activity of VEGFA.

## Introduction

Ewing sarcoma (EWS) is a kind of primitive neuroectodermal tumor, with higher occurrence in children and adolescents [1]. Although remarkable progress has been made in the treatment of EWS, its prognosis remains poor [1]. Lack of symptoms in early stages, high metastasis and chemotherapy refractory are main reasons [2]. Therefore, searching for the deep mechanisms is urged for therapeutic aims.

MicroRNAs (miRNAs) perform critical role in the initiation and development of many cancers. They are some short (22 bp) non-coding RNAs that mediate gene expression by binding to the complementary segment of target mRNAs [3]. MiRNAs exert important function in tumorigenesis, such as cell cycle progression, proliferation, epithelial to mesenchymal transition, and so on [4]. Several miRNAs have been reported to perform function during the tumorigenesis of EWS. Among these gene reported, miR-638 is a newly reported miRNA, whose biological function has not been unified in EWS yet. In colorectal carcinoma cells, miR-638 could suppress cell proliferation and epithelial to mesenchymal transition by targeting Sox-2 [5]. At the same time, Xia et al. also found miR-638 perform suppressive effects on the malignant phenotype of non-small-cell lung cancer cells (NSCLCs) [6]. While, a recent study confirmed that miR-638 enhanced the tumorigenic characteristics of melanoma cells *in vitro* [7], which means the putative effects of miR-638 might be different between different cell microenvironment. Herein, we will explore its expression and putative effects of miR-638 in EWS cells.

Angiogenesis is correlated with malignant phenotype of tumor, including chemotherapy resistance [8], proliferation, invasion, and metastasis. Recently, to investigate the molecular regulation of angiogenesis, a large number of genes associated with angiogenesis have been used as targets for the treatment of EWS, including fibroblast growth factor (FGF), insulin-like growth factor I receptor (IGF-IR), epidermal growth factor receptor (EGFR), CD31, and VEGF [9,10]. Among the vascular targeting agents, in particular, targeting VEGF have been evaluated in clinical trials [9]. Vascular endothelial cell growth factor A (VEGFA) was an important member of VEGF family, which reported to be a target gene of miR-638. Thus, we will further figure out whether it is involved in miR-638-mediated suppressive effects on EWS cells.

## Materials and methods

### Cell cultures

The human EWS cell lines RD-ES, SK-ES-1, and A673 were obtained from ATCC (American Type Culture Collection, Manassas, VA, USA). Human mesenchymal stem cells (MSCs) used in our experiments were obtained from normal adult human bone marrow withdrawn from bilateral punctures of the posterior iliac crests of three normal volunteers. MSCs were cultured at low confluence in IMDM, 10% FBS, and 10 ng/ml PDGF-BB (PeProtechEC). EWS cell lines were maintained in RPMI 1640 medium (Invitrogen Life Technologies, Carlsbad, CA, USA) supplemented with 10% fetal bovine serum (FBS) (PAA, Linz, Austria) with 100 mg/ml penicillin, and 100 mg/ml streptomycin (Invitrogen) at 37°C under 5% CO_2_.

### RNA extraction and quantitative

To determine the expression of miR-638 and target genes, the total RNA was obtained from EWS cells with a TRIzol reagent (Life Technologies, Darmstadt, Germany). To analyze miR-638 expression, total RNA was reversely transcribed using First-Strand cDNA Synthesis kit (Invitrogen). The specific stem-loop reverse transcription primers were as follows: miR-638-RT, 5′-GTCGTATCCAGTGCAGGGTCCGAGGTATTCGCACTG GAGGCCGCC-3′. The real-time PCR primer for U6 was U6-RT, 5′-AAAATATGGAACGCTTCACGAATTTG-3′. Quantitative real-time PCR was then performed using the Quanti-Tect SYBR Green PCR mixture on a CFX96TM Real-Time PCR Detection System (Bio-Rad, USA). U6 expression was served as internal control. The PCR primer sequences were used as follows: miR-638-F, 5′-AGGGATCGCGGGCGGGT-3′; miR-638-R, 5′-CAGTGCAGGGTCCGAGGT-3′; U6-F, 5′-CTCGCTTCGGCAGCACATATACT-3′; U6-R, 5′-ACGCTTCACGAATTTGCGTGTC-3′. To quantitate the mRNA expression of VEGFA, total RNA was reversely transcribed. The expression level of GAPDH was used as an internal control. The PCR primers were used as follows: VEGFA-F, 5′-GAAGGAGGAGGGCAGAATC-3′; VEGFA-R, 5′- CACACAGGATGGCTTGAAG-3′; GAPDH-F, 5′-TCAACGACCACTTTGTCAAGCTCA-3′; GAPDH-R, 5′- GCTGGTGGTCCAGGGGTCTTACT-3′. The relative expression level was calculated by 2-ΔΔCt methods, and the experiments were repeated three times.

### Western blot analysis

Samples were trypsinized and collected in ice-cold PBS after 48 h of transfection, RIPA buffer was used to isolate the total protein from the EWS cells. Protein concentrations from whole cell lysates were quantified by BCA assay Kit (Beyotime, Jiangsu, China). The protein (20–30 µg) were separated by SDS-polyacrylamide gelelectrophoresis (SDS-PAGE) and electro-transferred to polyvinylidene fluoride (PVDF) membranes (Millipore, USA). Then membranes were blocked by 5% non-fat dry milk and incubated overnight at 4°C in the presence of VEGFA (Cell Signaling Technology, USA), and GAPDH (ZSGB-BIO, Beijing, China). Upon washed in Tris-buffered saline-Tween 20 (TBST), the membranes were incubated in the presence of respective secondary antibody (ZSGB-BIO, Beijing, China). Proteins were visualized by chemiluminescence (ECL) kit (Millipore, USA) as recommended by the manufacturer. GAPDH was used as control.

### Plasmid construction

The coding sequences of VEGFA were amplified and inserted into pcDNA3.1 vector to generate pcDNA3.1-VEGFA plasmids, respectively. The PCR primer sequences were as follows: VEGFA-F: 5′-CCCAAGCTTCGCCGCCGCTCGGCGCCCG-3′, VEGFA-R: 5′-CCGGAATTCTCACCGCTCGG CTTGTCACA-3′, the correct PCR products were verified by sequencing (Genscript, Beijing, China). The empty pcDNA3.1 plasmids were used as negative control.

### Oligonucleotide transfection

MiR-638 mimic and scramble mimic oligonucleotides were obtained from Dharmacon (Austin, TX, USA). SK-ES-1 and RD-ES cells were transfected with the Dharmafect 1 (Dharmacon, USA) as recommended by the manufacturer. All medium was removed and replaced with fresh media after 6 h of transfection and grown for 48 h for the subsequent experiments.

### Luciferase reporter assay

The wild-type 3′-UTR sequence of VEGFA was generated from genomic DNA with the primer pairs VEGFA-UTR-F/R and cloned into the HindIII and NotI sites of the pGL-3 vector (Promega, USA). The mutated sequence was conducted with a QuickChange Site Directed Mutagenesis kit (Stratagene). The fragments were expressed as VEGFA_WT or VEGFA_MUT. EWS cell plated in 24-well plates at a density of 2 × 10^5^ per well for 24 h, were cotransfected with miR-638 mimic (40 nM/well) and the VEGFA_WT or VEGFA_MUT (40 ng/well) and pRL-TK Renilla luciferase reporter (10 ng/well) with the Lipofectamine 3000 (Invitrogen, USA). Renilla luciferase was performed as control. After 48 h post-transfection, luciferase activity was performed using the Dual Luciferase Reporter Assay System (Promega, USA). This experiment was repeated three times.

### Cell proliferation assay

The cell proliferation was analyzed using the Cell Counting Kit-8 (CCK-8; Dojindo, Japan). Approximately 5 × 10^3^ cells/well in 100 μl culture medium were plated in 96-well plates for 0, 24, 48 or 72 h. CCK-8 (10 µl) medium was added per well for 1.5 h at 37°C, and then the cell numbers measured by recording absorbance at 450 nm.

### Cell cycle assay

EWS cells in serum-free medium at 6 h after transfection, cells were serum starved for 24 h, then, all media was removed and replaced with fresh media for 48 h. The cells were collected and fixed in 75% ethanol for overnight at 4°C. After washed three times with PBS, RNase A was used at a concentration of 20 µg/ml in the dark at 37°C for 30 min. The cells were measured by flow cytometry (FACS) and completed within 30 min.

### Cell apoptosis analysis

At 48 h post-transfection, cells were harvested and resuspended in 1× binding buffer (BD Pharmingen, USA). The incidence of apoptosis was measured by with the Annexin V-FITC/7-AAD apoptosis detection kits (BD Pharmingen, USA) and then analyzed by FACS. The early apoptosis were represented by Annexin V staining, whereas the both Annexin V and 7-AAD contained late apoptotic cells.

### Tubule formation assay

The tube network formation assays were performed in EWS cell lines (SK-ES-1, RD-ES) under serum-free conditioned medium (high miR-638 expression group and control group). Matrigel (BD, Bedford, MA) for melting at 4°C for overnight, approximately 5 × 10^4^ cells per well were plated in 48-well plates coated with matrigel (100 μl) before incubating at 37°C for 30 min. The assay was found after 12 h and photographed with a light microscope. The number of crosses was analyzed by ImageJ software. All experiments were conducted in triplicate.

### Statistical analysis

The results were analyzed by GraphPad Prism 5 software, and the mean value was expressed as mean ± SEM. Two groups of data were analyzed by Student's *t*-test (two-tailed), unless otherwise indicated (*χ*^2^ test). *P*-values of less than 0.05 indicated statistical significance.

## Results

### The expression of miR-638 was suppressed in EWS cells

**Figure 1:**
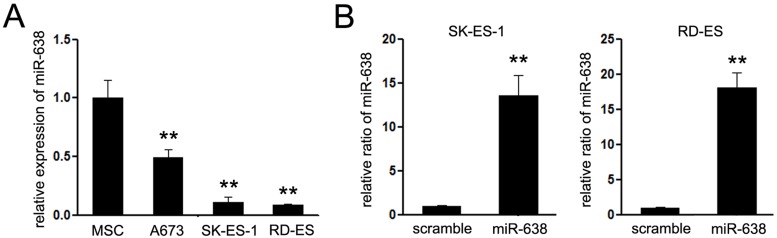
Down-regulation of miR-638 expression in EWS cell lines (**A**) Total RNA was isolated from MSC and EWS cell lines (A673, SK-ES-1, and RD-ES). Normalized miR-638 expression level detected by Quanti-Tect SYBR Green PCR Detection System. (**B**) SK-ES-1 and RD-ES cells were transfected with miR-638 mimic and collected. Transfection of miR-638 mimic significantly increased miR-638 expression in SK-ES-1 and RD-ES cells, as compared with transfected scramble cells. **P<0.01

To explore the putative role of miR-638 in EWS, the expression of miR-638 was firstly measured using Quanti-Tect SYBR Green PCR Detection System in several EWS cell lines, including A673, SK-ES-1, and RD-ES. Since the bone MSCs were reported to be the origin cell of EWS, MSCs were used as relative scramble group. As shown in Figure 1A, comparing with MSCs, EWS cells showed significantly lower expression of miR-638 (A673 for *P* = 0.0059, SK-ES-1 for *P* = 0.0006, and RD-ES for *P* = 0.0005 vs relative scramble groups). These results allegedly signify that miR-638 is down-regulated in EWS cell lines. Since the expression of miR-638 in SK-ES-1 and RD-ES cells than A673 cells, these two cells were chosen for subsequent experiments.

### MiR-638 inhibited cell growth and tube formation of EWS *in vitro*

**Figure 2:**
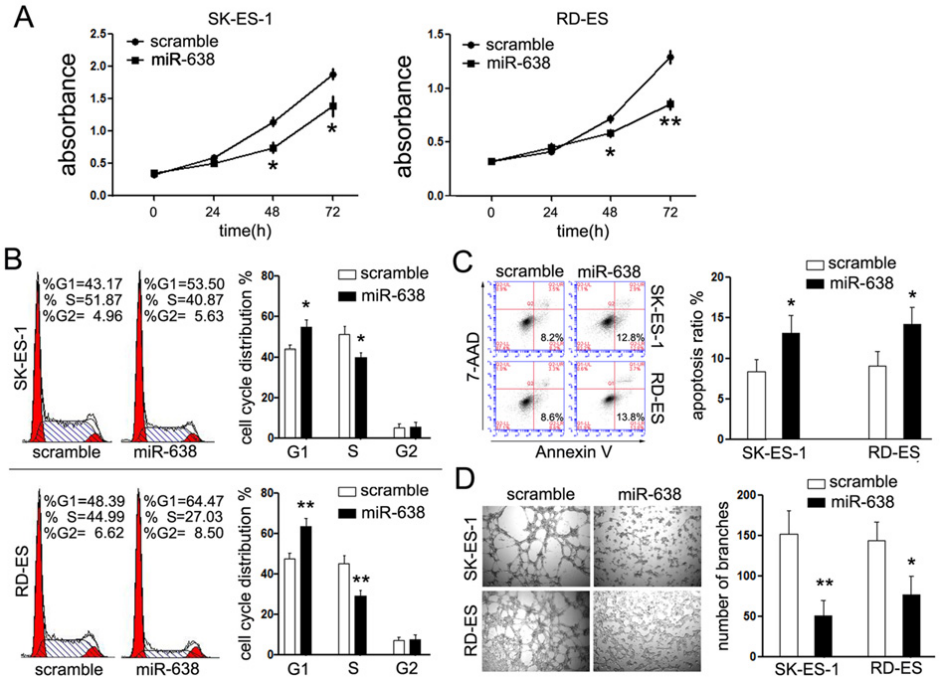
MiR-638 inhibits EWS cell growth and tube formation *in vitro* (**A**) The proliferation of EWS cell lines was analyzed by CCK-8 assays at every 24 h upon transfection for 3 days. (**B**) FACS analysis of cell cycle in cells transfected with scramble or miR-638 at 48 h after transfection showing more cells arrested in G1 phase in miR-638 transfected EWS cells. (**C**) FACS analysis of cell apoptosis rate after transfection at 48 h. The early apoptosis rate of overexpressed miR-638 SK-ES-1and RD-ES cells increased significantly, compared with control cells. (**D**) Capillary-like structure formation in EWS cell was examined by tube formation assays. SK-ES-1and RD-ES cells cultured on matrigel-coated plates, and tube formation assessed after 24 h. Representative images are shown (×100). The tube crosses was calculated using ImageJ software. *P<0.05; **P<0.01

To test the effects of miR-638 on the malignant phenotype of EWS cells, the expression of miR-638 was exogenously restored using artificial miR-638 mimic (Figure 1B). Companied with the overexpression of miR-638, the survival rates of EWS cells significantly suppressed (the *P*-values for 48 h were 0.018 in SK-ES-1 and 0.047 in RD-ES cells, and the *P*-values for 72 h were 0.0482 in SK-ES-1 and 0.0045 in RD-ES cells, relative to the scramble groups respectively) (Figure 2A). The results demonstrated that overexpression of miR-638 could obviously repress cell viability compared with controls in SK-ES-1 and RD-ES cells. Then, further research of the effects of miR-638 on EWS cells was performed. The effects of miR-638 on cell cycle were shown in Figure 2B. Exogenous expression of miR-638 in EWS cells revealed significant arrest of EWS cells in G0-G1 phase and obvious decline in S phase compared with controls. Concomitantly, the incidence of apoptosis was significantly higher in EWS cells with exogenous expression of miR-638 than the scramble groups (SK-ES-1, early apoptosis: 12.8% vs 8.2%, *P* = 0.0378; and RD-ES, early apoptosis 13.8% vs 8.6%, *P* = 0.0331) (Figure 2C).

The oxygen and nutrients of tumorigenesis depends on angiogenesis. To investigate the effects of miR-638 on the tube formation of EWS cells, tube-formation assays of RD-ES and SK-ES-1 cells were performed. Transfection with miR-638 significantly suppressed the tube-forming capacity of EWS cells compared with control cells, as the branches formed in EWS cells transfected with miR-638 was obviously decreased (SK-ES-1, branches formed: 152 vs 51, *P* = 0.0073; RD-ES, branches formed: 144 vs 77, *P* = 0.0234, relative to scramble groups) (Figure 2D).

### MiR-638 directly targets VEGFA in EWS

**Figure 3:**
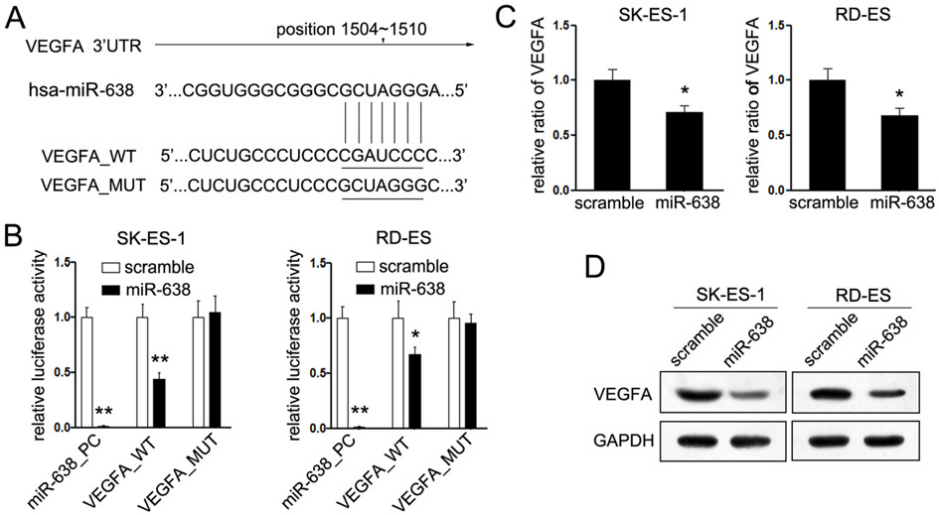
MiR-638 directly targets VEGFA (**A**) Schematic representation of the 3′UTR of VEGFA mRNA containing the putative miR-638 target site. (**B**) Relative luciferase assays comparing the SK-ES-1 and RD-ES cells transfected with pGL3-mutant (VEGFA_MUT) or pGL3-wild-type (VEGFA_WT) or untreated pGL3 vector (miR-638_PC). (**C**) The mRNA levels of VEGFA were measured by Quantitative RT-PCR in EWS cells after transfected with miR-638 or scramble mimic, respectively. (**D**) Protein levels of VEGFA and GAPDH were detected by Western blot in EWS cells after transfected with miR-638/scramble. *P< 0.05. **P < 0.01.

To underlying the molecular mechanisms of miR-638-mediated growth suppression in EWS cells, the bioinformatics analysis from three publicly available miRNA databases were used to search the candidate genes associated with miR-638. Among the candidate genes, our study focused on VEGFA for further experimental validation (Figure 3A). Firstly, the putative target role of VEGFA of miR-638 in EWS cells was identified using luciferase reporter assays. We found that the luciferase activity of the VEGFA_WT was notably suppressed after overexpression of miR-638 compared with scramble control, while the luciferase activity of the VEGFA_MT remained unaffected (Figure 3B) identifying that VEGFA was directly targeted by miR-638. As shown in Figure 3C,D, both the mRNA and protein levels of VEGFA were significantly down-regulated by miR-638 in EWS cell lines. Together, these findings strongly support that miR-638 regulates the expression of VEGFA in EWS cells transcriptionally.

### Overexpression of VEGFA reverses the suppressed effects of miR-638

**Figure 4:**
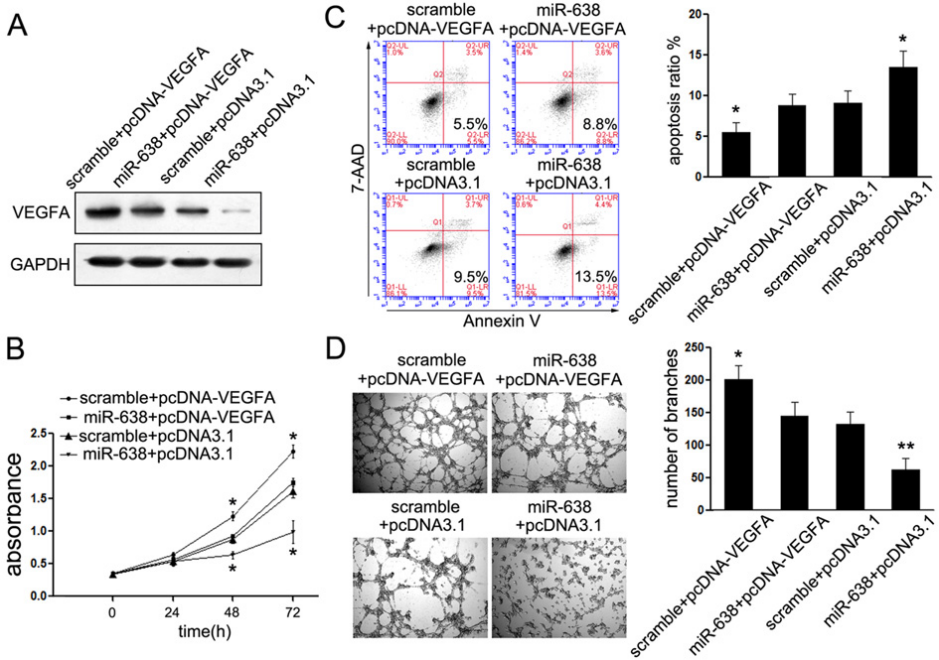
Overexpression of VEGFA reverses the suppressed effects of miR-638 (**A**) Cells overexpressing scramble or miR-638 were transfected with pcDNA3.1 or pcDNA-VEGFA. After 48 h, the expression levels of VEGFA protein were measured using Western blot assays. GAPDH was used as control. (**B**) The proliferation assays were conducted in SK-ES-1 cell overexpressing pcDNA3.1 or pcDNA-VEGFA with or without the miR-638 treatments. CCK8 assays were conducted at different time points. (**C**) Apoptosis in SK-ES-1 cells treated as described in (**B**). After 48 h of transfection, cell apoptotic rate was detected by FACS. (**D**). Tube formation in SK-ES-1 cells treated as described in (**B**), and assessed after 24 h. Representative images are shown (×100). The tube crosses was calculated using ImageJ software and the quantitative results are shown. *P< 0.05.

VEGFA is a potent angiogenic growth factor, commonly involved in the pathogenesis and progression of various cancers. To figure out whether it was also involved in the suppressive effects of miR-638 in EWS cells, the rescue assays were performed. As shown in Figure 4A, forced expression of VEGFA restored the VEGFA expression SK-ES-1 cells upon transfection with miR-638. Accompanied with restored expression of VEGFA, the suppressive effects on cell proliferation and encouraged cell apoptosis were partially abolished in SK-ES-1 cells after treatment with miR-638 (Figure 4B,C). In accordance with the hypothesis, we also found that exogenetic expression of VEGFA restored the suppressive effects of miR-638 on tube formation of SK-ES-1 cells (Figure 4D). These findings consistently suggested that VEGFA was involved in miR-638-mediated tumor suppressive effects in EWS cells.

## Discussion

Ewing sarcoma is the second most frequent tumor of bone and soft tissues in children and adolescents [1]. The mechanisms for tumorigenesis of EWS was less known. Herein, we found that miR-638 was suppressed in EWS cells and overexpression of miR-638 suppressed cell growth, induced cell apoptosis and inhibited tubule formation of EWS cells, which might be mediated through directly targeting VEGFA.

Dysregulation of miR-638 has been discovered in a cohort of tumors, including HCC [11], NSCLC [12], osteosarcoma [13], breast cancer [14], leukemia [15], melanoma [7], gastric cancer [16] as well as colorectal carcinoma [5,17]. For example, miR-638 regulates cell differentiation and proliferation by targeting cyclin-dependent kinase 2 (CDK-2) in leukemic cells [15]; and in BRCA1-deficient triple negative breast cancer tumors, miR-638 and miR-146 were suggested to perform as potential biomarkers for improved overall survival [14]. In this study, we firstly explored the putative effects of miR-638 in EWS cells. We found that the expression of miR-638 was significantly suppressed in EWS cell lines comparing with the MSCs, which were thought to be the origin cells of EWS. Restored the expression of miR-638 inhibited the proliferation rates of EWS cell lines, arrested the cell cycle progression and induced cell apoptosis of EWS cells consistently. These results suggested that miR-638 performs as a tumor suppressor in EWS.

Angiogenesis is vital for tumors and metastases, as they cannot grow in dimension greater than 2 mm without neo-angiogenesis [18]. Neo-angiogenesis provided the oxygen and nutrients for tumor growth and metastasis [19]. Before the formation of real tumor angio-network, tubule formation of tumor cells is one of the most specific methods for tumor cells to obtain oxygen and nutrients. Herein, we found that, other than the suppression on the malignant biological characteristics, significant suppression on the tube formation of EWS cells upon transfection with miR-638, which means miR-638 might participate in the regulation of neo-angiogenesis of EWS.

To figure out the mechanisms of miR-638-mediated tumor suppression, we searched for the putative target genes of miR-638. Among these genes, VEGFA attracted our attention mostly, as VEGF is a key regulator in response to angiogenesis during tumorigenesis [20]. Our study confirmed the target role of VEGFA by luciferase reporter assays. Treatment of miR-638 suppressed expression of both mRNA and protein level of VEGFA in EWS cells, and restored the expression of VEGFA could partially reversed the inhibitory effects of miR-638 in EWS cells could further identified the target role of VEGFA in EWS. These results suggested that VEGFA was a directly target gene of miR-638 in EWS.

In summary, our findings provide the first evidence that the expression of miR-638 significantly decreased and negative correlated to cell growth in EWS cell lines. Extraneous expression of miR-638 could suppress cell growth and tube formation by down-regulating VEGFA in EWS cell lines. This might provide invaluable information for EWS treatment study in the future.

## Abbreviations

EWS: Ewing sarcoma
miRNA: microRNA
MSC: mesenchymal stem cell
NSCLC: non-small-cell lung cancer cell
VEGFA: vascular endothelial cell growth factor A
